# Interactive effects of light, CO_2_ and temperature on growth and resource partitioning by the mixotrophic dinoflagellate, *Karlodinium veneficum*

**DOI:** 10.1371/journal.pone.0259161

**Published:** 2021-10-27

**Authors:** Kathryn J. Coyne, Lauren R. Salvitti, Alicia M. Mangum, Gulnihal Ozbay, Christopher R. Main, Zohreh M. Kouhanestani, Mark E. Warner

**Affiliations:** 1 College of Earth, Ocean, and Environment, University of Delaware, Lewes, Delaware, United States of America; 2 Department of Agriculture and Natural Resources, Delaware State University, Dover, Delaware, United States of America; 3 Department of Natural Resources and Environmental Control, Dover, Delaware, United States of America; 4 Department of Fisheries and Environmental Sciences, Gorgan University of Agricultural Science and Natural Resources, Gorgan, Golestan, Iran; CSIR-National Institute of Oceanography, INDIA

## Abstract

There is little information on the impacts of climate change on resource partitioning for mixotrophic phytoplankton. Here, we investigated the hypothesis that light interacts with temperature and CO_2_ to affect changes in growth and cellular carbon and nitrogen content of the mixotrophic dinoflagellate, *Karlodinium veneficum*, with increasing cellular carbon and nitrogen content under low light conditions and increased growth under high light conditions. Using a multifactorial design, the interactive effects of light, temperature and CO_2_ were investigated on *K*. *veneficum* at ambient temperature and CO_2_ levels (25°C, 375 ppm), high temperature (30°C, 375 ppm CO_2_), high CO_2_ (30°C, 750 ppm CO_2_), or a combination of both high temperature and CO_2_ (30°C, 750 ppm CO_2_) at low light intensities (LL: 70 μmol photons m^-2^ s^-2^) and light-saturated conditions (HL: 140 μmol photons m^-2^ s^-2^). Results revealed significant interactions between light and temperature for all parameters. Growth rates were not significantly different among LL treatments, but increased significantly with temperature or a combination of elevated temperature and CO_2_ under HL compared to ambient conditions. Particulate carbon and nitrogen content increased in response to temperature or a combination of elevated temperature and CO_2_ under LL conditions, but significantly decreased in HL cultures exposed to elevated temperature and/or CO_2_ compared to ambient conditions at HL. Significant increases in C:N ratios were observed only in the combined treatment under LL, suggesting a synergistic effect of temperature and CO_2_ on carbon assimilation, while increases in C:N under HL were driven only by an increase in CO_2_. Results indicate light-driven variations in growth and nutrient acquisition strategies for *K*. *veneficum* that may benefit this species under anticipated climate change conditions (elevated light, temperature and *p*CO_2_) while also affecting trophic transfer efficiency during blooms of this species.

## Introduction

Future oceans are projected to experience a substantial increase in the oceanic concentration of CO_2_ [1–3], as current CO_2_ partial pressure (*p*CO_2,_ 400 μatm) is estimated to reach 940 μatm by the end of twenty-first century [4]. This will be concurrent with rising atmospheric temperature which is predicted to increase 1.5 ^○^C above pre-industrial levels within the next few decades [1]. These anticipated changes will result in nutrient imbalance [5], along with increased stratification, and are expected to have a significant effect on phytoplankton from an individual species level such as growth and physiology [6, 7] to community levels including community composition [8, 9], trophic interactions [10], and dynamics [7, 11, 12]. In particular, many studies suggested that dinoflagellates, especially mixotrophic species, will have a competitive advantage under warming conditions [13, 14] because they are able to enhance autotrophic growth through predation [15–18].

Increasing temperature and CO_2_ can boost fundamental physiological processes and improve nutrient acquisition efficiency [19]. Since elevated CO_2_ reduces the energy needs for carbon assimilation, photosynthetic algae can reallocate the energy to other metabolic processes like growth [20–23]. For example, the growth rate of the dinoflagellate *Karenia brevis* significantly increased after exposure to high CO_2_ (1,000 ppm) for nine days [24]. The response, however, is not uniform in all taxa and reported to be species-specific regarding growth [8], chemical composition [7], and resource acquisition [25, 26]. While increasing temperature and CO_2_ stimulated the growth of the diatom, *Thalassiosira weissflogii*, *Dactyliosolen fragilissimus* exhibited a reduced growth rate [27]. Similarly, the cellular carbon and nitrogen content of *Heterosigma akashiwo* increased in response to combined high CO_2_ and temperature while the cellular stoichiometry of *Prorocentrum minimum* did not change significantly under the same treatment [28].

Light also plays a major role in regulating both growth rates and nutrient assimilation among phytoplankton species. Higher light exposure is expected with climate change due to increasing stratification and thermocline shoaling, as well as a decrease in dissolved organic matter allowing greater penetration of UV and photosynthetically active radiation [reviewed by 29]. While phytoplankton growth often increases until light is saturating, climate change conditions may interact with light to alter this response [30]. For example, optimal growth and carbon fixation in the coccolithophore *Gephyrocapsa oceanica* occurred at higher CO_2_ levels when cultured in limiting low light intensities compared to optimum light intensities [31]. A shift in the balance between light-responsive activities and metabolic activities, such as carbon and nitrogen assimilation, that respond to temperature and/or CO_2_ will likely affect phytoplankton in a species-specific manner. The interactive effects of multiple drivers, including light, temperature and *p*CO_2_ on the growth and nutrient assimilation of phytoplankton warrant further investigation to more accurately forecast effects of climate change on phytoplankton species and populations, including bloom-forming algae [7, 30, 32].

*Karlodinium veneficum* (synonymous with *K*. *micrum*, *Gymnodinium galatheanum*, *G*. *micrum*, and *Gyrodinium galatheanum*), is a member of the Dinophyceae [33] that forms blooms annually in the Delaware Inland Bays (DIBs) [34, 35] and other estuaries around the world [36]. *K*. *veneficum* grows autotrophically, but augments its growth through predation [36–38]. This species has received attention because of its ability to produce karlotoxins with hemolytic and cytotoxic properties [36, 39, 40], causing massive fish kills [41–44]. Research suggests that karlotoxin protects *K*. *veneficum* from predation [36], and also facilitates predation by *K*. *veneficum* [38, 45]. Fu et al. [46] also demonstrated an increase in toxicity of *K*. *veneficum* under elevated CO_2_ in concert with phosphate-limited or -replete conditions. Taken together, previous research on *K*. *veneficum* suggests that climate change-induced changes in toxicity may alter trophic interaction by this species. However, these interactions may also be affected by cellular nutrient status under anticipated changes in CO_2_, temperature and light. Carbon and nitrogen acquisition strategies by *K*. *veneficum* in response to the environmental stressors (CO_2_, temperature, light) have yet to be evaluated.

The study presented here investigated the interactive effects of light, CO_2_ and temperature on autotrophic growth and resource partitioning in *Karlodinium veneficum*. Given prior research results discussed above, one would expect that increases in *p*CO_2_ and temperature alone would increase cellular carbon and nitrogen due to enhanced metabolic activities and reduced energy required for carbon assimilation. When combined with higher light intensity, however, any increases in cellular carbon and nitrogen content may be offset or attenuated by an increase in growth rates, effectively dampening changes in cell-specific nutrient status. Using a multifactorial design, we tested this hypothesis—that an increase in *p*CO_2_ and temperature under low light conditions would increase cellular carbon and nitrogen assimilation while limiting growth, while an increase in *p*CO_2_ and temperature under high light conditions would enhance growth, while limiting cellular carbon and nitrogen assimilation. Results of this study indicated significant interactions between light and temperature on growth and resource partitioning for *K*. *veneficum*. These interactions may benefit this species while also affecting trophic transfer efficiency and phytoplankton community dynamics during blooms of *K*. *veneficum* under future climate change conditions.

## Materials and methods

### Culture maintenance

*Karlodinium veneficum* was originally isolated from Delaware Inland Bays, DE, and is available through the Provasoli-Guillard National Center for Marine Algae and Microbiota (https://ncma.bigelow.org/home; CCMP2936). Stock cultures were grown on a f/2-Si culture medium [47] made up in low nutrient 0.2 μm-filtered offshore seawater diluted to the salinity of 20 psu and autoclaved before addition of nutrients, trace metals, and vitamins. Batch cultures were acclimated at 25°C and 30°C for over 12 months, with a 12 h light: 12 h dark cycle, and transferred to fresh medium every 10–11 days. While cultures were not axenic, kanamycin (50 mg L^-1^) was added to cultures periodically to control bacterial growth.

### Experimental design

**Fig 1:**
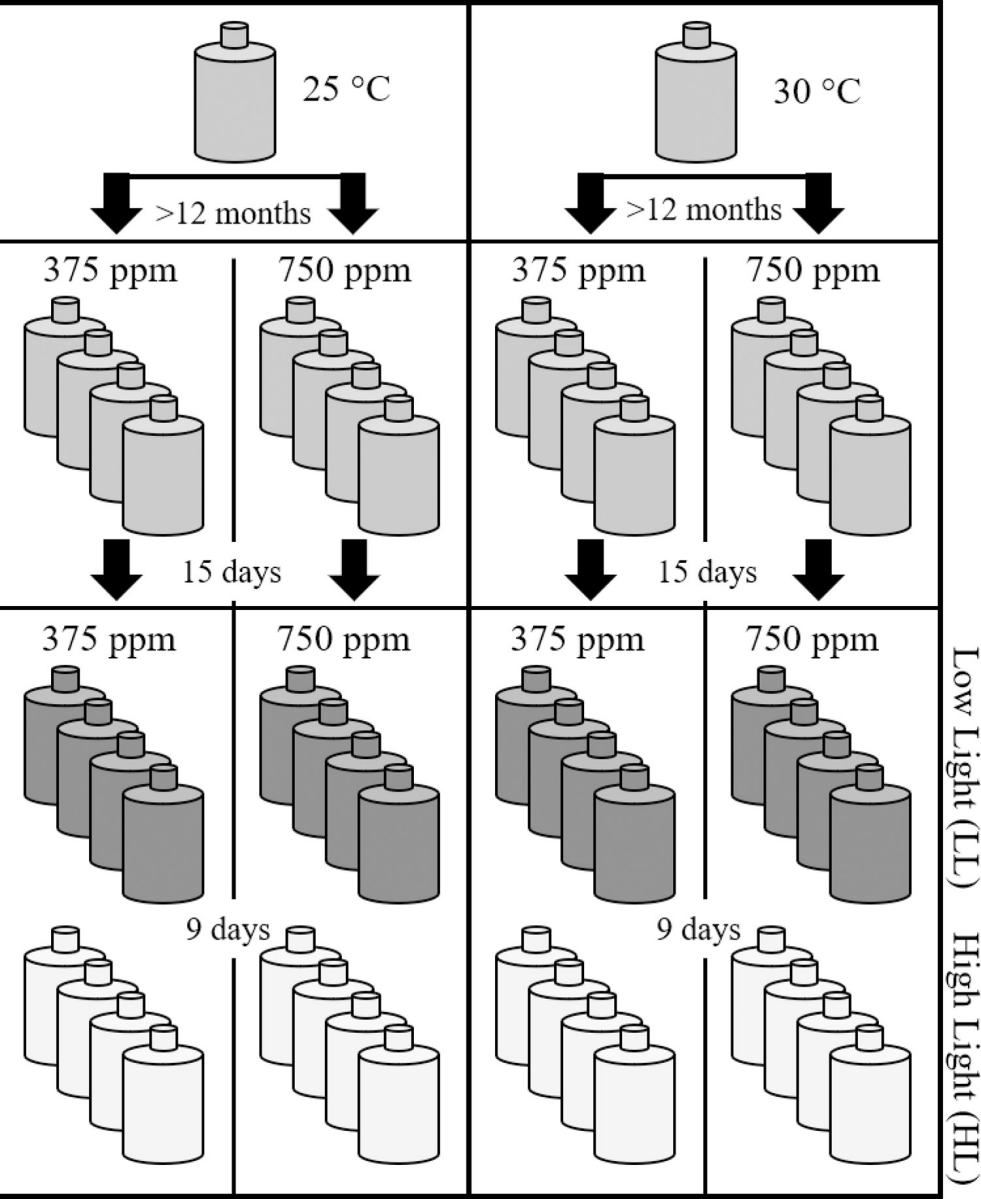
Schematic of CO_2_ and temperature treatments and incubation time for each treatment. See text for details.

Cultures (N = 4) were grown in 1-L polycarbonate bottles, fitted with silicone tubing attached to a glass-frit gas diffuser, and acclimated to gentle bubbling with air prior to the start of the experiment. Cultures at each experimental temperature were then gently bubbled with ambient (375 ppm) or elevated (750 ppm) CO_2_ in commercially prepared and certified air/CO_2_ gas mixture (Scott ^TM^ Gas Mixture, Scott Company, Plumsteadville, PA) for 15 days (at least four generations) before the start of the experiment. The gas was delivered through 0.2 μm air filters. For the low light (LL) and high light (HL) experiments, cultures were grown under cool white fluorescent lights at an irradiance of 70 (±2) μmol quanta m^-2^ s^-1^ and 140 (±3) μmol quanta m^-2^ s^-1^, respectively. At each light level, cultures were subjected to four treatment conditions: 375 ppm CO_2_ at 25°C, 375 ppm CO_2_ at 30°C, 750 ppm CO_2_ at 25°C, or 750 ppm CO_2_ at 30°C (Fig 1). Cultures (N = 4) were maintained in semi-continuous growth for an additional nine days by diluting cultures to initial cellular concentrations (50,000 cells mL^-1^) every other day with sterile f/2 medium, pre-equilibrated with air/CO_2_ mixture at the appropriate CO_2_ level.

### Growth rates and cell volume

Cells were fixed with gluteraldehyde at a final concentration of 0.1%, and counted using a hemocytometer (Hausser Scientific, Horsham, Pennsylvania). Growth (μ, d^-1^) was calculated as in Eq (1):

$$\mu =\ln\;\left(\frac{N_{2}}{N_{1}}\right)\cdot {\left(t_{1}-t_{2}\right)}^{-1}$$ (1)

where *N*_1_ and *N*_2_ were the cell abundance at times *t*_1_ and *t*_2_.

Cell size was measured on Day 9 for high light (HL) experiments only, using a Multisizer 3 Coulter Counter (Beckman Coulter, Inc., Brea, CA). Samples were diluted in filtered seawater and cell radius was calculated from the average of four measurements for each culture. Cell biovolume was calculated from the cell radius (r) as in Eq (2), assuming a spherical shape:

$$Volume=4\pi \;r^{3}/3$$ (2)

### pH and dissolved inorganic carbon

Samples for pH and DIC analysis were collected just prior to the start of the light cycle to minimize the effects of photosynthesis. The pH of each sample was analyzed using a Fisher Scientific AR15 Accumet Research pH meter, calibrated with NBS standards (Thermo Fisher Scientific, Inc., Waltham, MA). For DIC analysis, samples were collected from each replicate culture, placed in glass scintillation vials fitted with conical caps to remove any air space, preserved with 200 μL 5% HgCl_2_, and stored at 4°C until analysis within one week of sampling. Total DIC was determined by infrared gas analysis (Li-Cor Biosciences, Lincoln, NE) using the method of Friederich et al. [48]. Culture samples were compared to known DIC standards provided by the laboratory of Dr. A. Dickson (Scripps Institute of Oceanography). The value of CO_2_ was calculated from DIC and pH values using the CO2SYS software package (version 1.05; Upton, New York).

### Particulate carbon and nitrogen

Samples were collected on Day 9 and filtered onto pre-combusted (450°C for 4 hours) glass fiber filters for particulate carbon (PC) and nitrogen (PN) analysis. Filters were dried at 55°C and analyzed using a Costech Elemental Combustion System 4010 (Costech Analytical Technologies, Inc., Valencia, CA). EDTA and phenylalanine were used as standards.

### Carbon and nitrogen production rates

Particulate carbon (PC) and nitrogen (PN) production rates were calculated as in Eqs (3) and (4) [31]:

$$PC\;production\;rate=\mu (d^{-1})xPC(pg\;C\;{cell}^{-1})$$ (3)

$$PN\;production\;rate=\mu (d^{-1})xPN(pg\;N\;{cell}^{-1})$$ (4)

where μ is the growth rate and PC and PN are particulate carbon and nitrogen content per cell.

### RNA extractions and reverse transcriptase reactions

Samples were collected from each culture on Day 9 and filtered onto 3.0 μm polycarbonate membranes. Total RNA was extracted using the RNeasy Plant Mini Kit (Qiagen, Valencia, CA) and treated with DNase (Thermo Fisher Scientific Corp., Waltham, MA) to remove contaminating DNA. RNA was reverse transcribed in 20 μL reactions using SuperScript III First Strand Synthesis SuperMix Kit (Thermo Fisher Scientific) as described by Coyne et al. [49]. No-RT control reactions consisted of RNA that was subjected to DNase digestion, but not the first strand cDNA synthesis step to evaluate the presence of contaminating DNA in qPCR reactions.

### RuBisCO expression

#### Primer design

Primers targeting ribulose-1,5-bisphosphate carboxylase oxygenase (RuBisCO) large subunit (*rbcL*) and *β*-*actin* were designed for *K*. *veneficum* based on sequences available in GenBank (GenBank accession number AF463410 and AY345907, respectively). Primers were designed to amplify 250 bp of the *rbcL* gene and 207 bp of the endogenous control *β*-*actin* for *K*. *veneficum* (Table 1). To confirm the sequence of each gene amplified with these primers, DNA was extracted from *K*. *veneficum* as described in Coyne et al. [50], and amplified by PCR in 20 μL reactions. Each reaction contained 0.2 mM dNTPs, 0.5 μM primers (KvRUBISCO 508F and KvRUBISCO 758R or KvACTIN 703F and KvACTIN 910R; Table 1), 2.5 mM MgCl_2_, 10 μg μl^-1^ Bovine Serum Albumin (BSA), 1X Jump-Start Taq Polymerase Buffer (Sigma Chemical Company, St. Louis, Missouri) and 0.5 units Jump-Start Taq Polymerase (Sigma Chemical Company). Cycling parameters consisted of 34 cycles of 30 s at 94°C, 30 s at 55°C, and 1 min at 72°C, followed by a 5 min extension at 72°C. PCR products were cloned into pCR4 TOPO plasmid vector (Thermo Fisher Scientific). Clones for *K*. *veneficum rbcL* and *β-actin* were sequenced using Big Dye Terminator Sequencing Ready Reaction Kit on an ABI Prism 310 Genetic analyzer (Thermo Fisher Scientific).

**Table 1:** PCR primer sequences designed *K*. *veneficum*.

Target gene	Primer name	Sequence (5’-3’)
*rbcL*	KvRUBISCO 508F	GGGCCTGCAACAGGATT
	KvRUBISCO 758R	GTCCTTTAACCTCACCCG
*β-actin*	KvACTIN 703F	TGCCGAACGTGAGATTGT
	KvACTIN 910R	GCTGGCCTCCTTACCAAT

#### qPCR analysis

Transcript abundances for *rbcL* and *β-actin* were measured by qPCR in 10 μL reactions with 5 μL of SYBR Green Master Mix (Thermo Fisher Scientific), 0.3 μM of each primer, and 1 μL of template cDNA diluted 1: 20 with LoTE [3 mM Tris HCl (pH 7.5), 0.2 mM EDTA]. The cycling parameters for all reactions were 2 min at 50°C, 10 min at 95°C, followed by 40 cycles at 95°C for 30 seconds, an annealing step at 56°C for 30 seconds, and extension at 60°C for 1 minute. Products were evaluated by plotting the dissociation of duplex DNA with the stepwise increase in temperature from 60°C to 95°C. Transcript abundance was calculated by linear regression analysis, and *rbcL* transcript abundance was normalized to *β-actin* to determine the relative *rbcL* expression for each treatment.

### Statistics

Results for each analysis were compared using a one-way ANOVA, followed by Tukey HSD post hoc testing using PAST (PAlaeontological STatistics, ver.3; [51]. The data were evaluated for normality and homoscedasticity prior to statistical analysis. Differences were considered significantly different when p<0.05. Results for growth rates, particulate nitrogen and carbon analysis, productivity, and *rbcL* expression were also compared using a two-way ANOVA to assess significant interactions among combinations of light intensity, temperature and CO_2_ for all treatments and between temperature and CO_2_ within each light level.

## Results

### Dissolved inorganic carbon and CO_2_ concentrations

pH, *p*CO_2_ and DIC are shown in Table 2. Overall, pH was significantly lower at 750 ppm CO_2_ than at 350 ppm CO_2_ but there were no significant differences in pH between different light levels or temperatures. Dissolved inorganic carbon (DIC) concentrations were not significantly different between different light levels for each treatment. Under LL, DIC in the 30°C, 750 ppm CO_2_ treatment was significantly higher than the 350 ppm CO_2_ treatments. Under HL, the DIC concentration in the 25°C, 350 ppm CO_2_ treatment was significantly lower than other treatments (P<0.05). No significant differences were observed between CO_2_ levels within different CO_2_ treatments.

**Table 2:** pH, CO_2_ and DIC for each treatment (+/- standard deviation).

	pH		CO_2_ (ppm)		DIC (μM)	
Temperature	25°C	30°C	25°C	30°C	25°C	30°C
350 ppm LL	8.115 (0.212)	8.070 (0.073)	383 (178)	276 (56.6)	1276 (92.6)	1306 (26.3)
750 ppm LL	8.017 (0.232)	7.702 (0.030)	621 (212)	700 (56.0)	1326 (102)	1392 (16.7)
350 ppm HL	8.231 (0.129)	8.153 (0.059)	259 (98.9)	341 (53.6)	1198 (54.9)	1352 (178)
750 ppm HL	7.826 (0.068)	7.930 (0.113)	749 (78.0)	678 (40.0)	1370 (82.8)	1371 (30.2)

LL, low light; HL, high light.

### Growth rates

**Fig 2:**
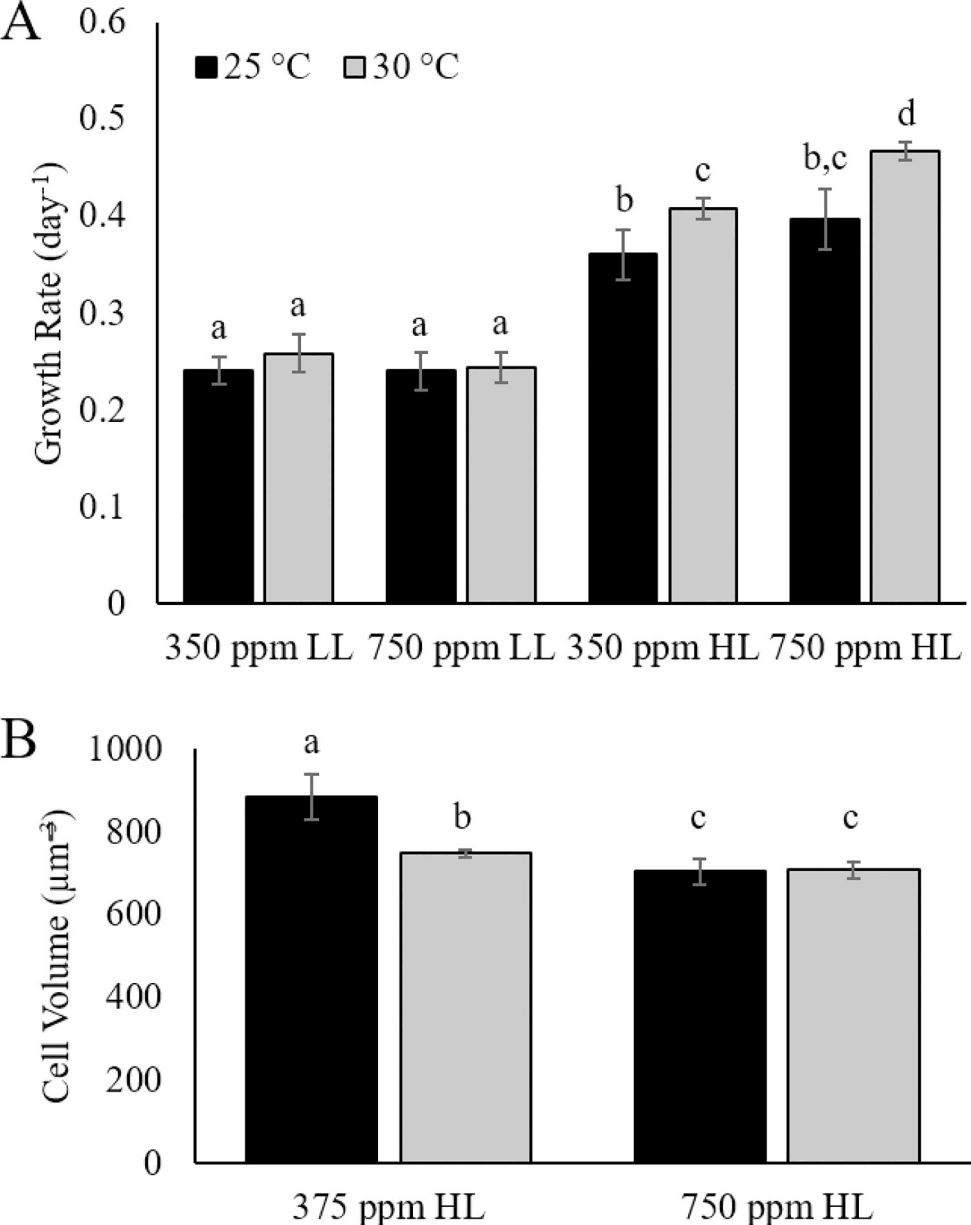
Growth rates and cell volumes. (A) Growth rate and (B) cell biovolume of *Karlodinium veneficum* in ambient (25°C) and elevated (30°C) temperature in combination with ambient (375 ppm) and elevated (750 ppm) CO_2_ under Low Light (LL; 70±2 μmol quanta m^-2^ s^-1^) and High Light (HL; 140±3 μmol quanta m^-2^ s^-1^) conditions. Error bars indicate the standard deviations of quadruplicate samples. Letters indicate significant differences (p<0.05).

For LL treatments, there were no significant differences in growth rates among any of the treatments with an average of 0.25 ± 0.01 day^-1^ (Fig 2A). Under HL conditions, growth rates were significantly higher than under LL, and ranged from 0.36 ± 0.03 day^-1^ to 0.47 ± 0.01 day^-1^ (Fig 2A). Growth rates were also significantly higher in 30°C treatments compared to ambient conditions at HL (p<0.05).

Analysis by two-way ANOVA revealed no significant interaction by CO_2_ and temperature with respect to growth either within light treatments or across both light treatments. However, there was a significant interaction between light and CO_2_ as well as light and temperature across all treatments (p<0.05).

Under HL conditions, elevated temperature and CO_2_, as well as the interaction of temperature with CO_2_, all had significant effects on the cell volume (p<0.005), shifting cell dimensions in favor of the smaller cells compared to those at ambient conditions of temperature and CO_2_ in HL (Fig 2B).

### Particulate carbon and nitrogen analysis

**Fig 3:**
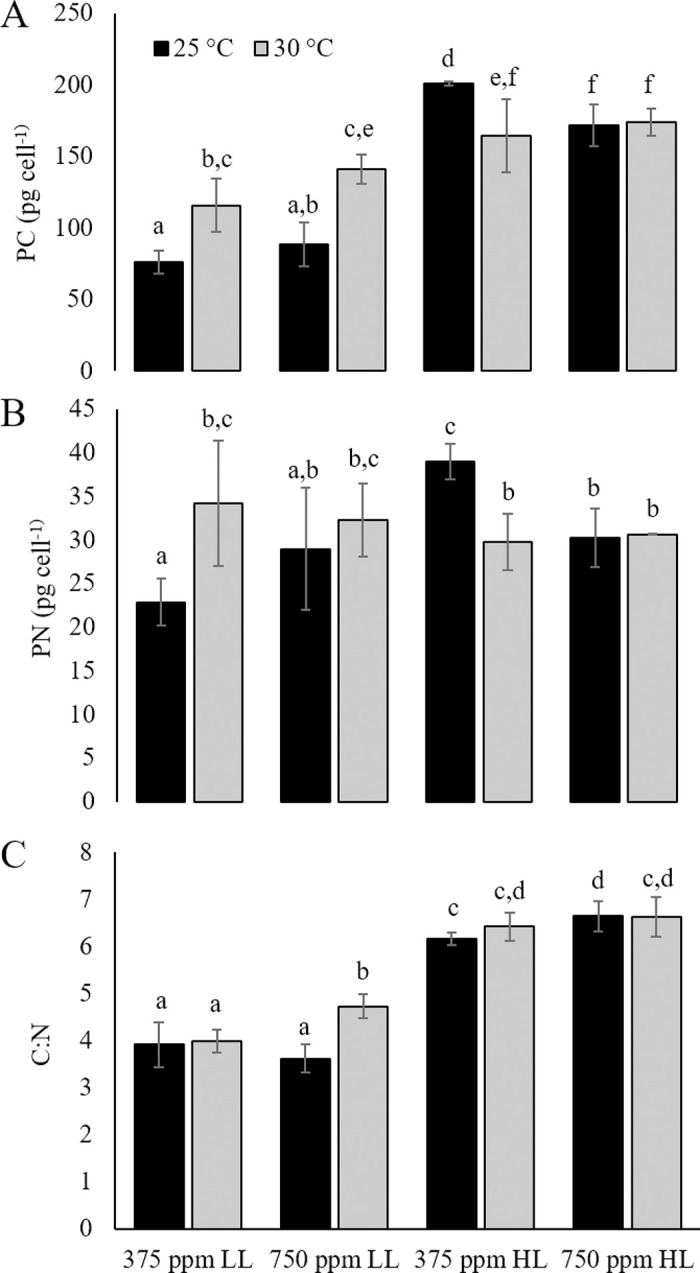
Particulate carbon and nitrogen. Cellular concentration of A) carbon (PC), B) nitrogen (PN), and C) carbon: nitrogen (C:N) of *Karlodinium veneficum* in ambient (25°C) and elevated (30°C) temperature in combination with ambient (375 ppm) and elevated (750 ppm) CO_2_ under Low Light (LL; 70±2 μmol quanta m^-2^ s^-1^) and High Light (HL; 140±3 μmol quanta m^-2^ s^-1^) conditions. Errors denote the standard deviations of quadruplicate samples. Letters indicate a significant difference between treatments (p<0.005).

Particulate carbon (PC) and nitrogen (PN) significantly increased in response to elevated temperature compared to ambient conditions under LL (p<0.00005, Fig 3A and 3B). Particulate carbon and nitrogen in HL were significantly higher than all LL treatments (p<0.05). In contrast to LL cultures, PC and PN were significantly higher in HL cultures maintained under ambient temperature and CO_2_ than in elevated temperature and/or CO_2_ under HL (p<0.05; Fig 3A and 3B).

Two-way ANOVA revealed a significant positive interaction between light and temperature for both PC and PN, but no significant interactions between light and CO_2_, or CO_2_ and temperature when all results were included. When each light level was evaluated separately, there was a significant interaction between temperature and CO_2_ on both PN and PC under HL but not LL.

Significant differences in carbon: nitrogen (C:N) under LL occurred only in the combined high temperature with high CO_2_ treatment (P<0.005) (Fig 3C). Two-way ANOVA analysis revealed a significant positive interaction between CO_2_ and temperature on cellular C:N under LL (p<0.001). C:N in HL cultures were significantly higher than LL cultures for all treatments (p<0.05; Fig 3C). CO_2_ also had a slight but significant positive effect on cellular C:N ratios under HL compared to other cultures at HL (Fig 3C). There were no significant interactions between temperature and CO_2_ for C:N within the HL treatment, and no significant interactions between light and CO_2_ or light and temperature across all treatments.

### Carbon and nitrogen production rates

**Fig 4:**
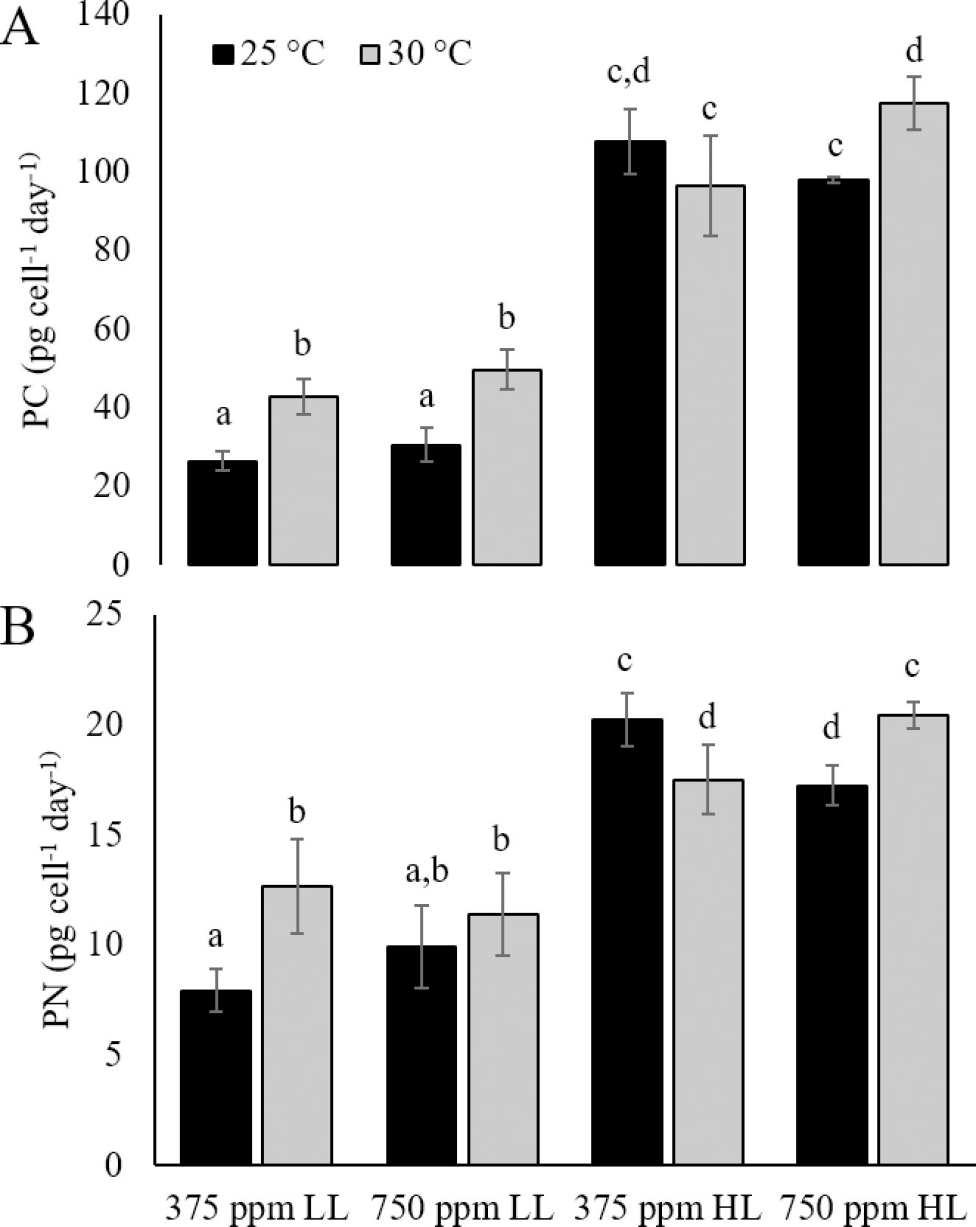
Rates of carbon and nitrogen production. Production rate of A) particulate carbon (PC) and B) particulate nitrogen (PN) in *Karlodinium veneficum* in ambient (25°C) and elevated (30°C) temperature in combination with ambient (375 ppm) and elevated (750 ppm) CO_2_ under Low Light (LL; 70±2 μmol quanta m^-2^ s^-1^) and High Light (HL; 140±3 μmol quanta m^-2^ s^-1^) conditions. Errors denote the standard deviations of quadruplicate samples. Letters indicate significant differences (p<0.005).

Particulate carbon (PC) and nitrogen (PN) production rates mirrored PC and PN in LL treatments, so that elevated temperature had a significant positive effect on carbon and nitrogen assimilation rates compared to cultures in ambient conditions under LL (p<0.01; Fig 4A and 4B). Under HL, carbon production rates were significantly higher in response to a combination of high temperature and CO_2_ (0>0.05; Fig 4A). Nitrogen production rates under HL significantly decreased in response to elevated temperature under ambient CO_2_ and under elevated CO_2_ under ambient temperature compared to cultures maintained at ambient temperature and CO_2_ or cultures maintained at elevated temperature and CO_2_ (Fig 4B).

Two-way ANOVA analysis revealed a significant interaction between temperature and light for both PC and PN production rates, but no significant interaction between light and CO_2_. Within HL treatments, there was also a significant interaction between CO_2_ and temperature for both PC and PN production rates (p<0.05).

### RuBisCO (*rbcL*) gene expression

**Fig 5:**
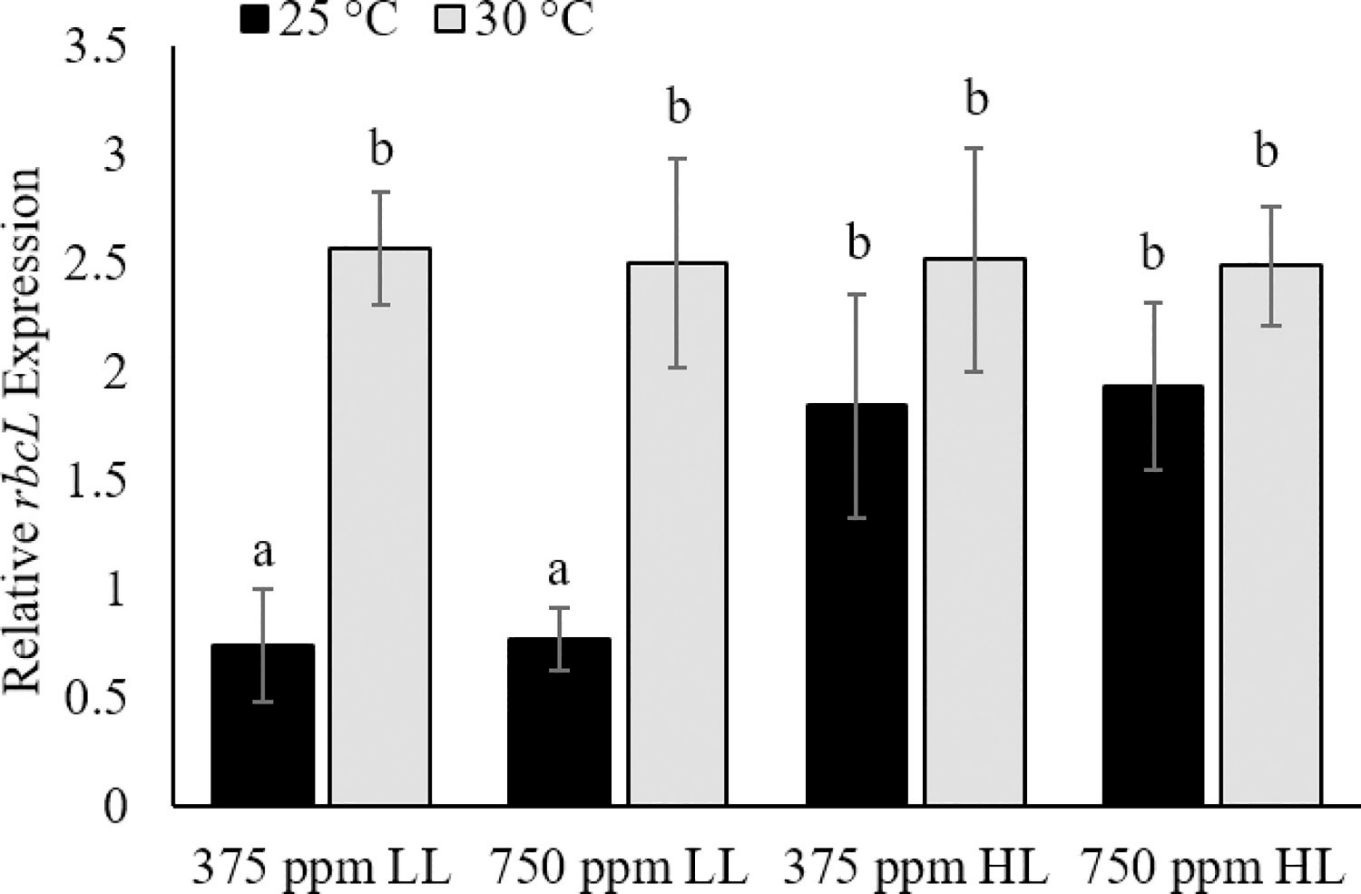
RuBisCO (*rbcL*) expression. Expression levels of *rbcL* in *Karlodinium veneficum* in ambient (25°C) and elevated (30°C) temperature in combination with ambient (375 ppm) and elevated (750 ppm) CO_2_ under Low Light (LL; 70±2 μmol quanta m^-2^ s^-1^) and High Light (HL; 140±3 μmol quanta m^-2^ s^-1^) conditions. Errors denote the standard deviations of quadruplicate samples. Letters indicate a significant difference from Control (p<0.05).

Transcript abundance of *rbcL* (relative to actin transcript abundance) was significantly higher in LL treatments under high temperature and a combination of high temperature and CO_2_ (p<0.05; Fig 5). Under HL conditions, however, no significant difference was detected among the treatments. Two factor ANOVA revealed a significant interaction between light and temperature (p<0.0005), but no significant interaction between light and CO_2_ or between CO_2_ and temperature within light levels or across all light levels.

## Discussion

**Fig 6:**
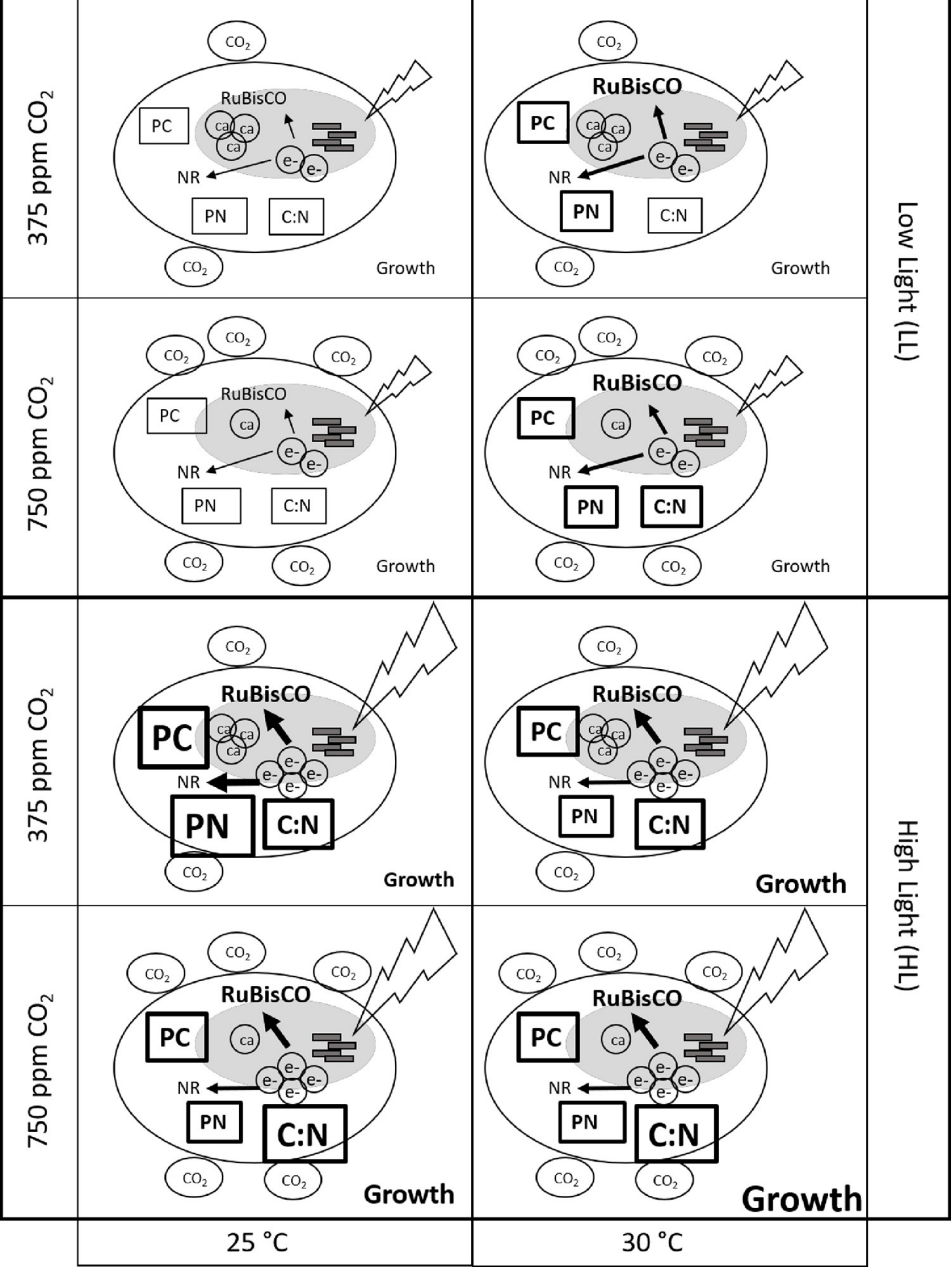
Illustration summarizing changes in measured parameters. Changes in particulate carbon and nitrogen, C:N ratios, growth and RuBisCO gene expression under ambient (375 ppm CO_2_, 25°C), high CO_2_ (750 ppm CO_2_, 25°C), high temperature (375 ppm CO_2_, 30°C), or combined high CO_2_ and high temperature (750 ppm CO_2_, 30°C) conditions in high light (HL) or low light (LL) intensities are shown. Increases in each parameter compared to ambient conditions under LL are indicated by bold font with further increases indicated by the size of the font. Darkened area within each cell represents the chloroplast. Ca, carbonic anhydrase; NR, nitrate reductase; e^-^, available reductant from light reactions.

This study investigated the interactive effects of light, temperature and CO_2_ on growth and cellular carbon and nitrogen assimilation for the mixotrophic dinoflagellate, *Karlodinium veneficum*. Several lines of evidence suggest that *K*. *veneficum* used different strategies for growth and resource partitioning under climate change conditions depending on light intensity. Comparison of cultures maintained under ambient temperature and CO_2_ showed that light alone had a significant impact, with higher growth rates, cellular carbon and nitrogen content and production rates and C:N at high light compared to low light conditions. Within each irradiance level, however, there were distinct differences in patterns of response to elevated CO_2_, elevated temperature and combined increases in CO_2_ and temperature treatments (Fig 6), with statistically significant interactions between light level and temperature for all parameters tested. This may be reflective of the need for cells to balance light-responsive activities such as light-harvesting and electron transport, with the effects of temperature and/or *p*CO_2_ on metabolic activities that serve as energy sinks, such as carbon and nitrogen assimilation [52, 53]. These changes in nutrient partitioning strategies of *K*. *veneficum* may have broader impacts on trophic transfer efficiency and phytoplankton community dynamics with anticipated changes in light regime under climate change conditions.

### Growth and resource partitioning under low light

Under low light conditions, there were no significant differences in growth rates among treatments (Fig 2A), suggesting that light was a limiting factor to population growth under all conditions tested. The addition of CO_2_ alone also had no significant effect on any of the parameters measured under low light conditions. Coastal environments such as those inhabited by *K*. *veneficum* experience wide fluctuations in CO_2_, with increases from terrestrial input and heterotrophic activities and decreases in CO_2_ from photosynthetic activity [21]. Dinoflagellates inhabiting these environments have efficient carbon concentrating mechanisms (CCMs; [54]) that increase CO_2_ concentration at the active site of RuBisCO through active transport of HCO_3_^-^.Under fluctuating CO_2_ concentrations such as those experienced in coastal environments, costly CCMs may be downregulated as molecular diffusion of aqueous CO_2_ meets the majority of the carbon demand. This investigation suggests that, with the increase in CO_2_ alone, any decline in CCMs had little effect on net carbon fixation or other metabolic activities under low light. Research on effects of increased CO_2_ in other phytoplankton species [e.g. 31], including dinoflagellates [28, 55] yielded similar results, with no changes in growth [28] or cellular carbon or nitrogen content under elevated CO_2_ [28, 55]. However, responses to elevated CO_2_ may differ even for the same species grown under different conditions [e.g. 56]. In contrast to results reported here, for example, Fu et al. [46] found that increased CO_2_ significantly enhanced growth of the same strain of *K*. *veneficum* used in this study. Light conditions as well as nutrient concentrations and nitrogen: phosphorus ratios used in Fu et al. [46] differed from those used here, making it difficult to directly compare results, and emphasize the complexity of climate change impacts on HABs involving multiple drivers [7, 30].

Increased particulate carbon (PC), particulate nitrogen (PN), and both PC and PN production rates by *K*. *veneficum* in response to elevated temperature suggest increased metabolic activity in cultures acclimated to 30°C. This is also supported by an increase in *rbcL* gene expression (Fig 5), providing evidence for enhanced RuBisCO activity and/or turnover in response to temperature [57]. The synergistic effects of elevated CO_2_ and temperature on PC indicate a substantial increase in carbon fixation as a result of combined temperature-induced increase in RuBisCO activity and an increase in CO_2_-saturated rate of carbon assimilation with the increased availability of CO_2_ substrate [58]. In contrast, there were no significant differences in PN among cultures exposed to high temperature, high CO_2_, or a combination of high temperature and CO_2_ under low light conditions. As nitrate was the only nitrogen source provided in these cultures, it would have been assimilated through nitrate reductase activity, which competes with RuBisCO for reductant generated by photosynthesis [59, 60]. Under low light, high temperature conditions, enhanced RuBisCO activity with elevated CO_2_ may have shifted the limited amount of reductant toward carbon fixation activities, at the expense of nitrogen assimilation. While cell size was not measured in low light cultures, it is reasonable to suggest that increased cellular PC and PN production rates in treatments at 30°C compared to ambient conditions would result in an increase in biovolume for *K*. *veneficum*, even without higher growth rates.

### Growth and resource partitioning under high light

While effects of elevated temperature and CO_2_ under low light conditions indicate an increase in productivity for *K*. *veneficum* without an increase in cell abundance, comparison to these same treatments at high light suggest that this species uses distinctly different strategies for resource partitioning when irradiance increases along with temperature and CO_2_. In contrast to low light, for example, resources were allocated to cell division at the expense of biomass when cultures were maintained at high light. Significant decreases in PC and PN on a per cell basis for cultures maintained in high temperature and/or CO_2_ suggested a potential decrease in cell size, which was confirmed by measurements of cell biovolume (Fig 2B). When calculated on a per biovolume basis, there were no significant differences in PC, PN or C:N across all treatments at high light, indicating a size dependency on carbon and nitrogen assimilation when light was not limiting.

Of particular interest here is the interaction between temperature and CO_2_ under high light conditions with respect to cellular carbon and nitrogen production rates (Fig 4). Results presented here suggest that (1) when compared to cultures under ambient conditions of temperature and CO_2_, the decrease in PC and PN content for high temperature and CO_2_ treatments under HL can be attributed to decreases in cell biovolume, whereas (2) the significant increase in PC and PN production rates in cultures maintained with a combination of high temperature and CO_2_ implies that productivity was limited by low temperature at high CO_2_ and by low CO_2_ at high temperature. Studies on coccolithophores have demonstrated a link between *p*CO_2_ optima for carbon fixation and temperature [61]. Results of this study suggest a similar shift in *p*CO_2_ optima for *K*. *veneficum*, so that when light was not limiting, a combination of increased CO_2_ with increased temperature was necessary to achieve significant gains in growth and productivity compared to each individual driver.

### Relevance to natural populations of *Karlodinium veneficum*

Expanded seasonal warming [62] along with increased toxicity in *K*. *veneficum* with elevated CO_2_ [46] may have devastating consequences for local fisheries, as blooms become more toxic and occur over a wider temporal and geographic range. The temperatures used in this study spanned a range that *K*. *veneficum* would encounter in mid-Atlantic environments, where blooms of this species occur annually [34, 35]. Previous analysis of field data for Delaware’s inland bays, however, indicated that the abundance of natural *K*. *veneficum* populations peaked at temperatures between 25°C and 29°C, with a sharp drop in cell density at temperatures used here for high temperature treatments (30°C) [35]. The field data is consistent with reports of laboratory culture experiments using the same strain of *K*. *veneficum*, in which Vidyarathna et al. [35] identified a temperature optimum for this species of 28.6°C with a drop in growth rate at 30°C. The ability of *K*. *veneficum* to maintain growth rates after long-term exposure to elevated temperature (> 1 year) in the present study suggests a capacity for either phenotypic acclimation or genetic adaptation to conditions [53, 63, 64] that may benefit *K*. *veneficum* under anticipated future temperature conditions.

The reduction in cell biovolume for *K*. *veneficum* maintained in high light under elevated temperature and/or CO_2_ conditions may also improve its competitive ability under climate change conditions. Smaller cells can more efficiently harvest light and nutrients, and are better able to maintain their position in the water column [65, 66]. It has been predicted that species with smaller cell volumes will become dominant under climate-induced stresses such as light-saturated conditions along with high temperature, and elevated CO_2_ [67, 68]. The decrease in cell biovolume and increase in growth rates for *K*. *veneficum* measured here for high light cultures in response to high temperature and CO_2_ may provide this species with a competitive advantage under climate change conditions, and suggests an increase in intensity for *K*. *veneficum* blooms.

### Trophic implications

In addition to abiotic factors such as light, *p*CO_2_ and temperature regimes encountered in the natural environment, trophic interactions also have an effect on *K*. *veneficum* population dynamics and may be altered by anticipated changes in climate.

Zooplankton play a key role in regulating phytoplankton abundance and community composition through top-down control [69, 70]. Grazing effects are modulated by physical parameters such as temperature and light [71], as well as nutrient concentrations [72–74], taxonomic composition [75, 76] and the quantity and nutritional quality of prey [77, 78]. In addition, a shift in phytoplankton toward HAB species can negatively affect both the growth and grazing activity of microzooplankton [38].

Results of this study point toward potential impacts of climate change on trophic transfer efficiency by *K*. *veneficum* as both predator and as prey [42, 79]. Increased toxicity of *K*. *veneficum* as would be expected under elevated CO_2_ [42], for example, may reduce top down control on this species by meso- and microzooplankton predators [41, 80] while increasing predation by *K*. *veneficum* on other protists [38, 79]. For example, mixed prey experiments described in Adolf et al. [41] showed that the presence of toxic *K*. *veneficum* inhibited grazing by the heterotrophic dinoflagellate, *Oxyrrhis marina*, on co-occurring non-toxic strains. Effects of *K*. *veneficum* toxicity on copepod grazers has also been shown to be species-dependent [81], suggesting that an increase in toxicity along with increased intensity of *K*. *veneficum* blooms under elevated *p*CO_2_ and high light conditions as demonstrated here might skew copepod populations, favoring more tolerant species. Nutrient cycling dynamics may also be affected. For example, Saba et al. [82] reported an increase in DOM released by the copepod grazer, *Acartia tonsa*, when feeding on toxic *K*. *veneficum* vs. a non-toxic strain, suggesting a feedback loop that may contribute to an increase in toxic blooms of this species [82].

In addition to changes in toxicity, altered nutritional quality of *K*. *veneficum* under climate change conditions may have a negative effect on trophic transfer. Carbon: nitrogen ratios (C:N) are considered a proxy for cellular protein content, with a higher ratio indicating lower nutritional value for zooplankton [8, 83]. Even when phytoplankton biomass was reported to increase in response to climate change conditions, the low nutritional quality of the phytoplankton (high C:N) negatively affected higher trophic levels, reducing mass transfer efficiency [84] and enhancing the stoichiometric mismatch between phytoplankton and their consumers [83]. Zooplankton abundance and community composition may also be negatively impacted by high C:N, with a decrease in production [85] and hatchability [86]. Relative to results reported here, Smith [87] also observed significantly reduced egg production and hatching success when *Acartia tonsa* grazed on *K*. *veneficum* under elevated temperature and *p*CO_2_ treatments. Hence, the trend toward increased C:N in *K*. *veneficum* suggests that blooms of this species under anticipated climate change conditions will have a negative effect on zooplankton grazers, in spite of the potential increase in the availability of algal prey due to higher growth rates.

## Conclusions

Results of this study highlight differential responses to anticipated changes in temperature, CO_2_, and light intensity for the mixotrophic dinoflagellate, *K*. *veneficum*. Growth rate was enhanced under high light in response to both elevated temperature and a combination of high temperature and CO_2_, suggesting an increase in the intensity of blooms of this species with climate change. The increase in C:N for the elevated temperature and CO_2_ treatments may also impact trophic interactions, with a reduction in nutritional value for zooplankton grazing on *K*. *veneficum*. When coupled with the increase in toxicity [46], results presented here indicate that top-down control of *K*. *veneficum* blooms by predators may decrease, providing a competitive advantage to this species under anticipated climate change conditions.
